# Antimicrobial and anti-inflammatory properties of Maranta arundinacea extract against Campylobacter jejuni and Campylobacter coli in T84 cells

**DOI:** 10.1099/mic.0.001658

**Published:** 2026-01-14

**Authors:** Banaz Star-Shirko, Nicolae Corcionivoschi, Ozan Gundogdu

**Affiliations:** 1 Faculty of Infectious and Tropical Diseases, London School of Hygiene and Tropical Medicine, London, UK; 2 Agri-Food and Biosciences Institute, Food Microbiology, Newforge Lane, Belfast, UK

**Keywords:** anti-inflammatory, *Campylobacter coli*, *Campylobacter jejuni*, invasion, plant antimicrobial compounds, T84

## Abstract

*Campylobacter*, a bacterium commonly found in the chicken gut, is the leading cause of bacterial foodborne gastroenteritis globally. Despite various interventions aimed at controlling *Campylobacter* in the food chain, such as enhanced biosecurity measures, improved hygiene practices and farm-level controls, reducing its prevalence remains a significant challenge. While the European Union’s (EU) 2006 ban on antimicrobials as growth promoters was primarily intended to control antimicrobial resistance, its impact on *Campylobacter* load has been limited. The emergence of antibiotic-resistant *Campylobacter* has created a requirement to develop alternative methods to improve food safety, enhance performance and mitigate pathogenic bacteria. This study explored the potential of *Maranta arundinacea* (arrowroot) extract as a prospective dietary supplement for both humans and chickens. The investigation focused on its safety, its ability to reduce *Campylobacter* in T84 intestinal epithelial cells and its anti-inflammatory properties. Results showed that 4% and 25% concentrations of arrowroot extract were non-cytotoxic to human T84 cells and significantly reduced bacterial growth in *Campylobacter jejuni* strains. Additionally, the extract inhibited the growth of *Campylobacter coli* strains and *Escherichia coli*, with statistical significance observed against *E. coli* at the 25% concentration. These results suggest that arrowroot extract could be a promising natural alternative for addressing antibiotic resistance and enhancing food safety.

## Introduction

Natural antimicrobial compounds have been proven to be effective and novel antimicrobial agents. These compounds are derived from plants, animal metabolites or micro-organisms [1]. Exploring new antimicrobials is important for controlling multidrug-resistant strains of pathogenic micro-organisms. The World Health Organization (WHO) promotes the use of medicinal herbs as remedies to support the lack of conventional treatment [2]. One of the major causes of bacteria developing resistance is the use of antibiotics in the food industry, for example, for growth performance [3,6]. However, it is well known that antibiotic use can lead to the potential generation of antibiotic-resistant pathogenic bacteria. Therefore, the routine supplementation of antibiotics in poultry feed as growth enhancers has been reduced [7,8]. The ban on antimicrobial growth promoters in 2006 by the European Union (EU) has created a requirement to develop alternative methods to improve performance and potentially reduce the numbers of pathogenic bacteria [9,10]. In addition to farm biosecurity measures, incorporating multiple interventions can enhance food safety along the meat production chain. By employing diverse antimicrobial strategies with different modes of action, synergistic effects can be achieved, reducing bacterial contamination and improving the safety of the final product [11,15]. Despite the widespread use of control measures, including hygiene protocols, biosecurity in farming, chemical feed additives, vaccinations, passive immunization, competitive exclusion cultures, host genetic selection strategies, bacteriophage therapy and bacteriocin applications, such strategies have largely failed to effectively reduce the presence of pathogenic bacteria, such as *Campylobacter* strains, in the food chain [16].

*Campylobacter* is present within the chicken gut and is the leading cause of bacterial foodborne gastroenteritis in humans worldwide [17,18]. The economic burden of *Campylobacter* infection is severe [19,20]. The impact of *Campylobacter jejuni* infection extends beyond the treatment of diarrhoeal illness, as infection can lead to secondary complications, such as Guillain–Barré syndrome and stunted growth in children from low-resource areas [21]. As poultry demand continues to grow, maintaining the health of chickens and minimizing *Campylobacter* presence are essential to reducing human infection risks. Natural antimicrobials present a promising solution for combating pathogenic bacteria in poultry production. These substances, derived from plants, micro-organisms or other natural sources, can help inhibit bacterial growth while potentially reducing reliance on conventional antibiotics. Beyond their antimicrobial properties, natural alternatives may offer additional benefits, such as improving gut health, enhancing immune function and contributing to overall animal welfare. By integrating these compounds into farming practices, producers could develop safer and more sustainable methods for controlling harmful bacteria while meeting consumer demands for healthier poultry products. New antimicrobials are important for controlling multidrug-resistant strains of *Campylobacter*. As an example, the efficacy of Auranta 3001 (a mixture of organic acids and plant extracts) has been shown to reduce the ability of *C. jejuni* and *Campylobacter coli* to invade human T84 cells *in vitro*. It was shown that this antimicrobial agent has a direct effect on *C. jejuni* and *C. coli* invasion of intestinal epithelial cells (IECs) [22,23]. Natural antimicrobials have been used as a viable alternative for pathogen reduction [24]. Previous studies have shown that arrowroot contains fructo-oligosaccharides, which can be used to obtain higher biomasses of probiotics [25]. In addition, the highly digestible property of arrowroot makes it particularly useful for infants, the elderly, patients with digestive issues and those with coeliac disease [26]. Furthermore, the antioxidant activity of the phenolic compounds of arrowroot plants can be utilized and developed for potential use in controlling *Campylobacter*. In general, prebiotics appear to be effective in poultry and reduce the colonization of foodborne pathogens, such as *Salmonella,* in the gastrointestinal tract of poultry [27,28].

Natural antimicrobials present a vast reservoir of potential therapeutic options; however, only a limited number have been explored for their anti-*Campylobacter* properties [29]. Arrowroot contains a diverse array of biologically active phytochemicals, notably flavonoids and terpenoids, which have demonstrated antibacterial properties in several studies [30]. Flavonoids exert antimicrobial effects through multiple mechanisms, such as inhibition of nucleic acid synthesis, disruption of cytoplasmic membrane integrity and function and interference with energy metabolism.

Additionally, flavonoids may form complexes with bacterial proteins via nonspecific interactions, such as hydrogen bonding and hydrophobic effects, or through covalent bonding, further contributing to their antibacterial activity [31,32].

The application of natural antimicrobials at specific stages of the chicken lifecycle to reduce *Campylobacter* pathogens remains uncertain. Our study aimed to investigate the use of arrowroot extract as a potential dietary supplement for both humans and chickens. Specifically, we aimed to explore arrowroot’s antimicrobial ability to inhibit the growth of *Campylobacter* and its anti-inflammatory properties. To achieve this, we examined the impact of arrowroot extract on the growth performance of *Campylobacter* strains, with the goal of using it as a chicken feed supplement to reduce *Campylobacter* levels.

## Methods

### Bacterial strains and growth conditions

*C. jejuni* and *C. coli* WT strains used in this study are listed in Table 1. For general growth, all *C. jejuni* and *C. coli* strains were grown on Columbia Blood Agar (CBA) plates (Oxoid, UK) supplemented with 7% (v/v) horse blood (TCS Microbiology, UK) and the *Campylobacter* selective supplement Skirrow (Oxoid) at 37 °C under microaerobic conditions (10% Carbon dioxide (CO_2_), 5% Oxygen (O_2_) and 85% Nitrogen (N_2_) (Don Whitley Scientific, UK). The *Escherichia coli* strain was grown on CBA plates (Oxoid, UK) supplemented with 7% (v/v) horse blood (TCS Microbiology, UK) at 37 °C under microaerobic conditions (10% CO_2_, 5% O_2_ and 85% N_2_) (Don Whitley Scientific, UK).

**Table 1.** Bacterial strains used in this study

Strain	Description	Source/reference
11168H	A hypermotile variant of the NCTC 11168 WT human clinical isolate strain	[58]
81–176	A WT human clinical isolate from an outbreak of acute gastroenteritis following the consumption of raw, contaminated milk	[59]
RC039	WT chicken isolate from Northern Ireland	[60]
*C. coli* M8 strain	Isolated from retail chicken meat in Vietnam	[61]
*C. coli* C75 strain	Isolated from farmed chicken faeces samples in Vietnam	[61]
*E. coli* DH5α strain	Chemically competent, commercially available strain used for all assays	New England BioLabs (NEB; UK)

### Materials

*Maranta arundinacea* (arrowroot) extract was obtained from Holland and Barrett, UK. Arrowroot extract was serially diluted in MilliQ water from 50% to 1% (w/v). Each dilution was vortexed thoroughly to ensure homogeneity. The samples were then incubated under continuous shaking at 500 r.p.m. for 24 h at room temperature. Following incubation, the solutions were subjected to sterile filtration using a 0.22 µm membrane filter to remove particulates and ensure sterility.

### Susceptibility to arrowroot

Broth dilutions were performed to determine the minimum inhibitory concentartion (MIC) of arrowroot extract and the lowest concentration that resulted in bacterial death Minimum bactericidal concentration (MBC). Twofold dilutions of the antimicrobial agent in a liquid growth medium, dispensed in a 96-well microtitration plate (microdilution), were performed. Individual overnight bacterial cultures were harvested by centrifugation, washed with PBS and diluted to ~1×10^6^ c.f.u. ml^−1^ in Müeller–Hinton broth (MHB). Each well was inoculated with ~5×10^5^ c.f.u. ml^−1^ of this bacterial culture (final concentration). After mixing, the 96-well microtitration plate was incubated at 37 °C under microaerobic conditions (10% CO_2_, 5% O_2_ and 85% N_2_), following the methodology published by the European Society of Clinical Microbiology European Committee on Antimicrobial Susceptibility Testing 2019 (EUCAST, 2019). Arrowroot extract was diluted (50% down to 1% w/v) in MilliQ water, thoroughly vortexed and left for 24 h shaking at 500 r.p.m. Several strains of *C. jejuni, C. coli* and *E. coli* were used (see Table 1), along with positive controls (untreated, inoculated broth media) and negative controls (broth media). Growth curves were monitored by measuring OD at 600 nm (OD_600_) using a microplate reader. To account for turbidity or colour contributed by the test extracts, parallel wells containing medium plus extract but no cells were included as blanks. The absorbance values from these blanks were subtracted from the corresponding treatment wells prior to analysis. This ensured that the reported OD_600_ values reflected only microbial growth and not background interference from the extracts.

### Human IEC culture

T84 cells (ECACC 88021101) were obtained from the European Collection of Authenticated Cell Cultures (ECACC). T84 cells were cultured in a 1:1 mixture of Dulbecco’s modified Eagle’s medium and Ham’s F-12 medium (DMEM/F-12; Thermo Fisher Scientific, USA) supplemented with 10% FBS (Labtech, UK), 1% nonessential amino acid (Sigma-Aldrich, USA) and 1% penicillin–streptomycin (Sigma-Aldrich). T84 cells were cultured at 37 °C in a 5% CO_2_-humidified environment. For infection assays, DMEM/F-12 without penicillin–streptomycin was used. DMEM/F-12 without phenol red was used for the lactate dehydrogenase (LDH) assay. Cell lines were grown at 37 °C in a 5% CO_2_ incubator (Sanyo, USA).

### *C. jejuni* interaction, invasion and intracellular survival assays

T84 cells (≈10^6^ cells) in a 24-well plate were washed with pre-warmed PBS three times, and a bacterial suspension with an appropriate OD_600_ of 0.2 in complete growth medium without penicillin–streptomycin was prepared. T84 cells were then infected with 1 ml of bacterial suspension [multiplicity of infection (MOI) of 200:1] and incubated for 3 or 24 h at 37 °C in a 5% CO_2_ incubator. In some experiments, T84 cells were pretreated with 4% or 25% for 3 h, washed three times with PBS and then infected with the bacteria. For the interaction (adhesion and invasion) assays, infected T84 cells were washed three times with pre-warmed PBS to remove unbound extracellular bacteria. Then, 500 µl of 0.1% (v/v) Triton X-100 (Sigma-Aldrich) was added into each well and incubated for 20 min at room temperature. After incubation, each well was pipetted for 1 min to lyse the IECs. The lysates were then briefly vortexed, and ten-fold serial dilutions were performed in a 96-well plate. Ten microlitres of each dilution was plated onto CBA plates, and the plates were incubated for 48 h at 37 °C under microaerobic conditions. The number of *C. jejuni* interacting with IECs was enumerated. An additional step of treatment with gentamicin (150 µg ml^−1^) for 2 h at 37 °C in a 5% CO_2_ incubator to kill extracellular bacteria was performed prior to lysis for invasion assays. After incubation with gentamicin, T84 cells were washed three times with pre-warmed PBS. The cells were lysed with 0.1% (v/v) Triton X-100, and the lysates were diluted in PBS and plated onto CBA plates as described above.

For intracellular survival assays, after infection with *C. jejuni* for 3 h, T84 cells were incubated with gentamicin (150 µg ml^−1^) for 2 h at 37 °C in a 5% CO_2_ incubator to kill extracellular bacteria, followed by a further 18 h incubation with gentamicin (10 µg ml^−1^) at 37 °C in a 5% CO_2_ incubator. IECs were lysed as described above, and fivefold serial dilution was performed, plated onto blood agar plates for c.f.u. enumeration.

#### Investigations of the interaction between *C. jejuni* strain 81-176 and pretreated T84 cells with arrowroot extract

**Fig. 1.**
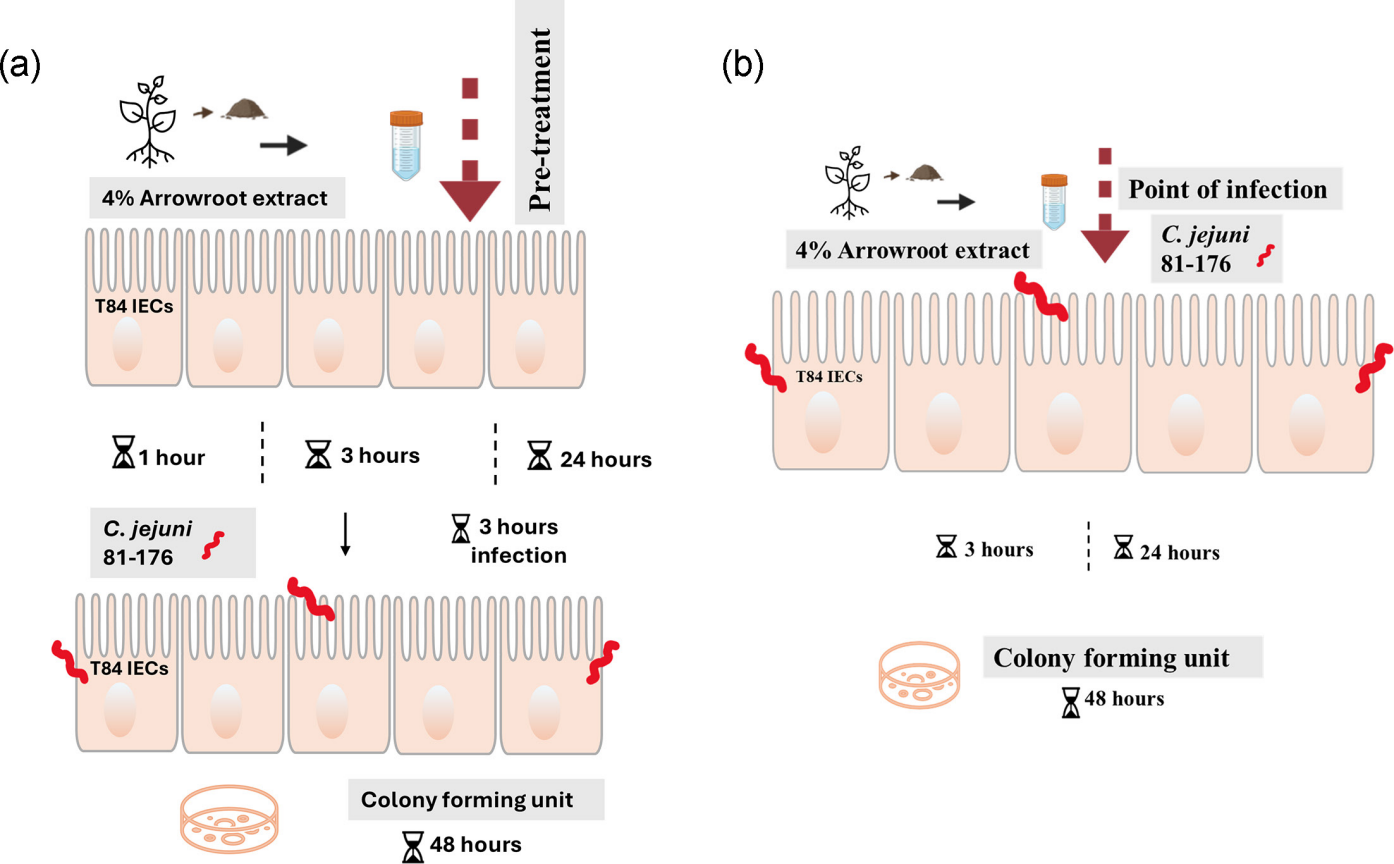
Overview of study design. (**a**) Pre-treatment with arrowroot and infection of T84 cells. (**b**) Treatment with arrowroot at POI of T84 cells.

Here, we investigated the host–pathogen interaction of *C. jejuni* strain 81–176 with T84 cells. We studied the time-dependent adhesion, invasion and survival of this strain at a concentration of 4%. T84 cells were pretreated with arrowroot for 1, 3 and 24 h, after which cells were washed with PBS and infected with *C. jejuni* strain 81–176 (OD_600_=0.2; MOI of 200:1) and incubated for 3 h at 37 °C in a 5% CO_2_ incubator (Fig. 1a). For interaction assays, T84 cells were then washed with PBS and lysed with 0.1% (v/v) Triton X-100, and c.f.u. per millilitre were recorded after 48 h of incubation. For invasion assays, T84 cells were incubated with gentamicin (150 µg ml^−1^) for 2 h to kill extracellular bacteria and then lysed with 0.1% (v/v) Triton X-100, and c.f.u. per millilitre were recorded after incubation. For intracellular survival assays, after infection with *C. jejuni* for 3 h, T84 cells were incubated with gentamicin (150 µg ml^−1^) for 2 h at 37 °C in a 5% CO_2_ incubator to kill extracellular bacteria, followed by a further 18 h incubation with gentamicin (10 µg ml^−1^) at 37 °C in a 5% CO_2_ incubator.

#### Investigations of the interaction between *C. jejuni* and T84 cells with the addition of arrowroot extract at the point of infection

Here, we investigated the host–pathogen interaction of *C. jejuni* strain 81–176 with T84 cells. We studied the time-dependent adhesion, invasion and survival of this strain at a concentration of 4%. T84 cells were introduced to arrowroot at the point of infection (POI) with *C. jejuni* strain 81–176 (OD_600_=0.2; MOI of 200:1) for 3 and 24 h at 37 °C in a 5% CO_2_ incubator (Fig. 1b). For interaction assays, T84 cells were washed with PBS and lysed with 0.1% (v/v) Triton X-100, and c.f.u. per millilitre were recorded after 48 h of incubation. For invasion assays, T84 cells were incubated with gentamicin (150 µg ml^−1^) for 2 h to kill extracellular bacteria and then lysed with 0.1% (v/v) Triton X-100, and c.f.u. per millilitre were recorded after 48 h of incubation. For intracellular survival assays, after infection with *C. jejuni* for 3 and 24 h, T84 cells were incubated with gentamicin (150 µg ml^−1^) for 2 h at 37 °C in a 5% CO_2_ incubator to kill extracellular bacteria, followed by a further 18 h incubation with gentamicin (10 µg ml^−1^) at 37 °C in a 5% CO_2_ incubator.

#### Interleukin-8 ELISA

Here, interleukin-8 (IL-8) induction in T84 cells by *C. jejuni* strain 81–176 was investigated by measuring the expression patterns of the cytokine IL-8 after 3 and 24 h of exposure to 4% arrowroot at the time of infection with *C. jejuni* strain 81–176 (OD_600_=0.2). Human IL-8 ELISA was performed using an uncoated ELISA kit (Invitrogen, USA) as described in Section 2.6 to determine whether *C. jejuni* strain 81–176 interaction and invasion correlate with *C. jejuni*-induced IL-8 secretion by T84 cells.

### Investigations of the anti-inflammatory property of arrowroot at the selected concentrations

The pro-inflammatory response of T84 cells was investigated by measuring the expression patterns of the cytokine IL-8 released from T84 cells infected for 3 h with various bacterial strains (*C. jejuni* 11168H, 81–176, RC039, *C. coli* C75, M8 and *E. coli*). Subsequently, the T84 cells were treated with 4% and 25% concentrations of arrowroot for 3 h, using the cell culture supernatant and a Human IL-8 Uncoated ELISA kit (Invitrogen, USA). The kit was used according to the manufacturer’s instructions. T84 cells were infected for 3 h as described before in Section 2.5, with the supernatant collected for the assay and supernatant from uninfected T84 cells used as controls. After infection, the supernatants were collected and transferred into 1.5 ml microcentrifuge 72 tubes and either kept on ice and used straight away or snap-frozen and stored at −80 °C before further analysis. ELISA assays were conducted using Nunc™ MaxiSorp™ 96-well flat-bottom microplates (Invitrogen). Initially, the wells of the plate were coated with 100 µl of the capture antibody (anti-human IL-8) diluted 1:250 in coating buffer and incubated overnight at 4 °C. The next day, the plate was washed three times with 260 µl per well wash buffer composed of 0.05% (v/v) Tween 20 (Sigma-Aldrich) in PBS, whilst samples were thawed gently on ice before analysis. To block the wells, 200 µl of 1X ELISA/ELISAPOT diluent was added to the well before the plate was incubated for 1 h at room temperature whilst shaking. The buffer was removed, and the wells were washed once with wash buffer. The standard was reconstituted in distilled water and used with 1X ELISA/ELISAPOT diluent to perform twofold serial dilutions in the designated wells according to the kit’s protocol. Additionally, 100 µl of 1X ELISA/ELISAPOT diluent was loaded into wells to be used as a blank. The thawed samples were vortexed, and 100 µl of each sample was added to the appropriate wells. The plate was covered with aluminium foil and allowed to incubate for 2 h at room temperature whilst shaking. After incubation, the wells were washed with wash buffer four times and then incubated with 100 µl per well of detection antibody (diluted 1:250 in ELISA/ELISPOT diluent) for a further hour with shaking at room temperature. The wells of the plate were washed four times with wash buffer prior to being incubated with Avidin-horseradish peroxidase at room temperature for 30 min with shaking. The plate was washed five times with wash buffer, then 100 µl per well of 1X Tetramethylbenzidine (TMB) solution was added, the plate was sealed, and incubated at room temperature for 15 min with shaking. Hundred microlitres of Stop Solution (2 N Sulfuric acid (H_2_SO_4)_; Sigma-Aldrich) was added into each well, and plates were immediately read at absorbances of 570 and 450 nm using a SpectraMax M3 Multi-Mode microplate Reader and SOFTmax Pro 7 software. For analysis, the 570 nm values were subtracted from the 450 nm values to adjust for optical plate imperfections, with the values from the standard used to create an eight-point standard curve for calibration.

### LDH cytotoxicity assay

CyQUANT™ LDH Cytotoxicity Assay (Thermo Scientific) was used to investigate the effect of chemical treatments on the viability of T84 cells. T84 cells (≈10^4^ cells per well) were plated in triplicate wells in a clear-bottom and black 96-well plate (Corning, USA), followed by incubation at 37 °C in a 5% CO_2_ atmosphere for 18 h. Complete growth medium was replaced with DMEM/F-12 without phenol red, supplemented with 5% (v/v) FBS. Ten microlitres of DEPC-treated water (Invitrogen, USA) was added to one set of triplicate wells of cells for spontaneous LDH activity controls. For chemical-treated samples, chemicals were diluted in 10 µl of DEPC-treated water to give the final concentration. For maximum LDH activity controls, nothing was added at this step. The plate was incubated at 37 °C in a 5% CO_2_ incubator for 3 h. After treatment with 4% and 25% arrowroot extract, 10 µl of 10X Lysis Buffer was added to the set of triplicate wells of maximum LDH activity controls and mixed gently by tapping, followed by incubation at 37 °C with 5% CO_2_ for 45 min. After incubation, 50 µl of medium from each well was transferred to a new clear-bottom and 44 black 96-well plate. Then, 50 µl of reaction mixture was added to each sample and gently mixed. The plate was then incubated at room temperature for 30 min, protected from light. Fifty microlitres of Stop Solution was then added to each well and mixed gently by tapping. The absorbance at both 490 and 680 nm was measured using a SpectraMax M3 Multi-Mode Microplate Reader (Molecular Devices, USA). The LDH activity was measured by subtracting the absorbance at 680 nm from the absorbance at 490 nm. Cytotoxicity (%) was calculated using the equation below:

.$$\%\;Cytotoxicity=\frac{Chemical\;traeted\;LDH\;activity-Spontaneous\;LDH\;activity}{Maximum\;LDH\;activity-Spontaneous\;LDH\;activity}\times 100$$

### Trypan blue exclusion assay

To measure the viability of T84 cells under experimental conditions, a trypan blue exclusion assay was performed. After the desired incubation under experimental conditions, 300 µl of 0.25% (w/v) trypsin-EDTA was added into each well, and the plates were incubated for 10 min at 37 °C in a 5% CO_2_ incubator. After the cells were detached, 1 ml of complete culture medium was added into each well and pipetted to mix well. Fifty microlitres of cell suspension was transferred to 1.5 ml microcentrifuge tubes and mixed with 50 µl of 0.4% (w/v) trypan blue solution. Twenty microlitres of cell suspension with trypan blue solution was loaded onto the haemocytometer. The number of viable and dead cells was counted in four outer squares. Percentage viability was calculated based on the enumerated cells.

### Statistical analysis and graphing

At least three biological replicates were performed in all experiments. Each biological replicate was performed in three technical replicates. For statistical analysis and graphing, GraphPad Prism 10 for Windows (GraphPad Software, USA) was used. Unpaired t-tests were used to compare two datasets for significance, with * indicating *P*<0.05, ** indicating *P*<0.01, *** indicating *P*<0.001 and **** indicating *P*<0.0001.

## Results

### Identification of the MICs

**Fig. 2.**
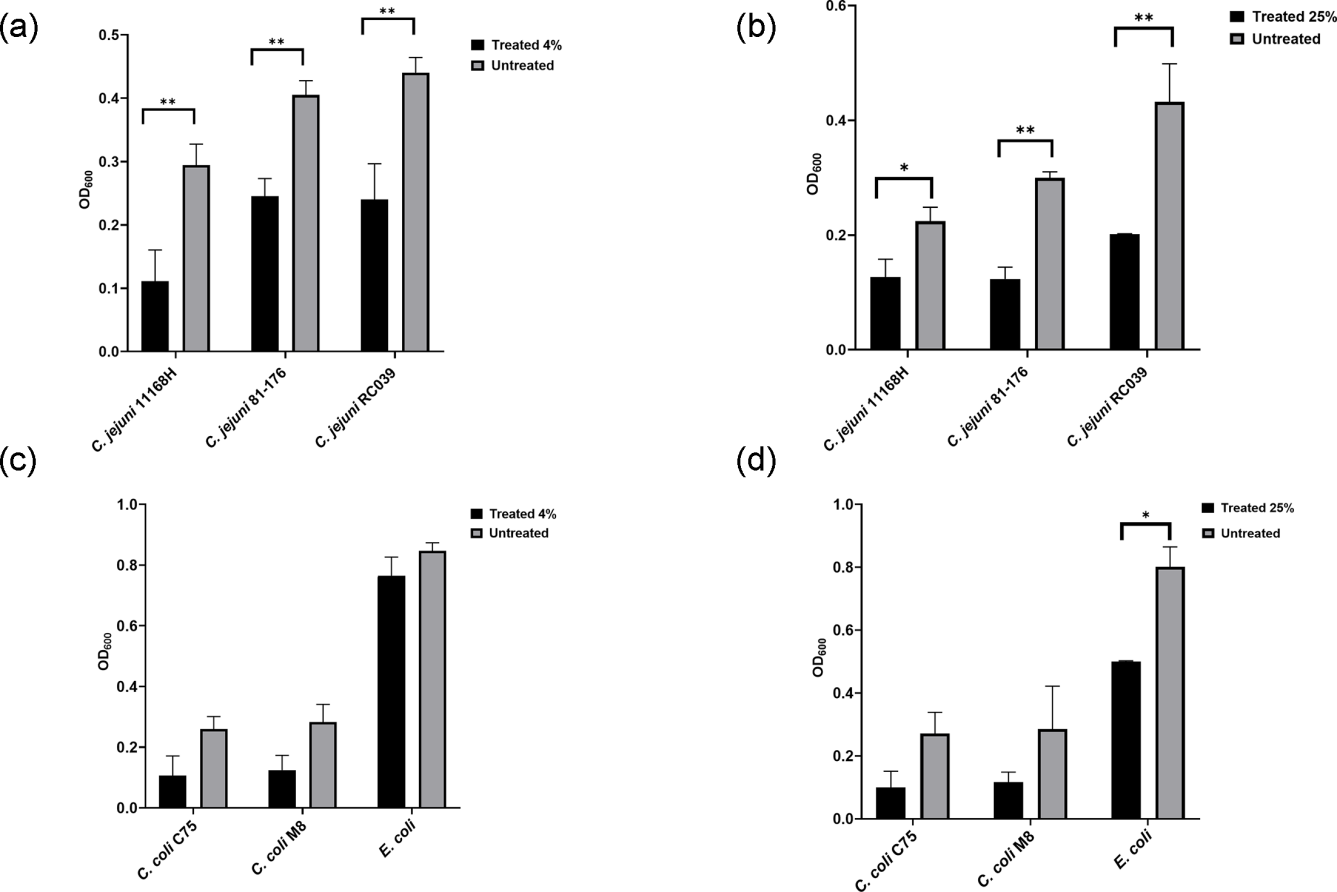
The inhibitory concentration of arrowroot extract at 4% and 25% concentrations against *C. jejuni*, *C. coli* strains and *E. coli* 11168H in MHB incubated for 48 h. (**a**) Significant inhibition of growth of *C. jejuni* 11168H, 81–176 and RC039 (** = P<0.01) at 48 h incubation control at 4% concentration of arrowroot extract compared with the control. (**b**) Significant inhibition of growth of *C. jejuni* 11168H and 81–176 (** = P<0.01) and RC039 (* = P<0.05) at 48 h incubation at 25% concentration of arrowroot extract. (**c**) Inhibition of the growth of *C. coli* C75, M8 and *E. coli*. (**d**) *E. coli* statistical significance reduction against *E. coli* at 25% concentration. Experiments were repeated in three biological and three technical replicates. Unpaired t-tests were used to compare two datasets for significance. Data are presented as mean±sem.

To characterize the effect of arrowroot on *C. jejuni* pathogenicity, we first attempted to identify the MICs for all isolates (*C. jejuni* 11168H, 81–176, RC039, *C. coli* C75, M8 and *E. coli*). While we were unable to determine the exact MICs, arrowroot concentrations of 4% and 25% were effective in reducing bacterial growth (Fig. 2). A 4% concentration of arrowroot extract significantly inhibited the growth of *C. jejuni* 11168H, 81–176 and RC039 (** = *P*<0.01) after 48 h of incubation compared with the control. At 25% concentration, the extract significantly inhibited the growth of *C. jejuni* 11168H and 81–176 (** = P<0.01) as well as RC039 (* = P<0.05) under the same conditions. Additionally, arrowroot extract was effective in reducing the growth of *C. coli* C75, M8 and *E. coli*, though statistical significance was observed only against *E. coli* at the 25% concentration.

Based on these findings, we concluded that concentrations of 4% and 25% are most appropriate for further investigations, as they effectively reduce bacterial growth.

### *C. jejuni* interaction, invasion and intracellular survival assays

**Fig. 3.**
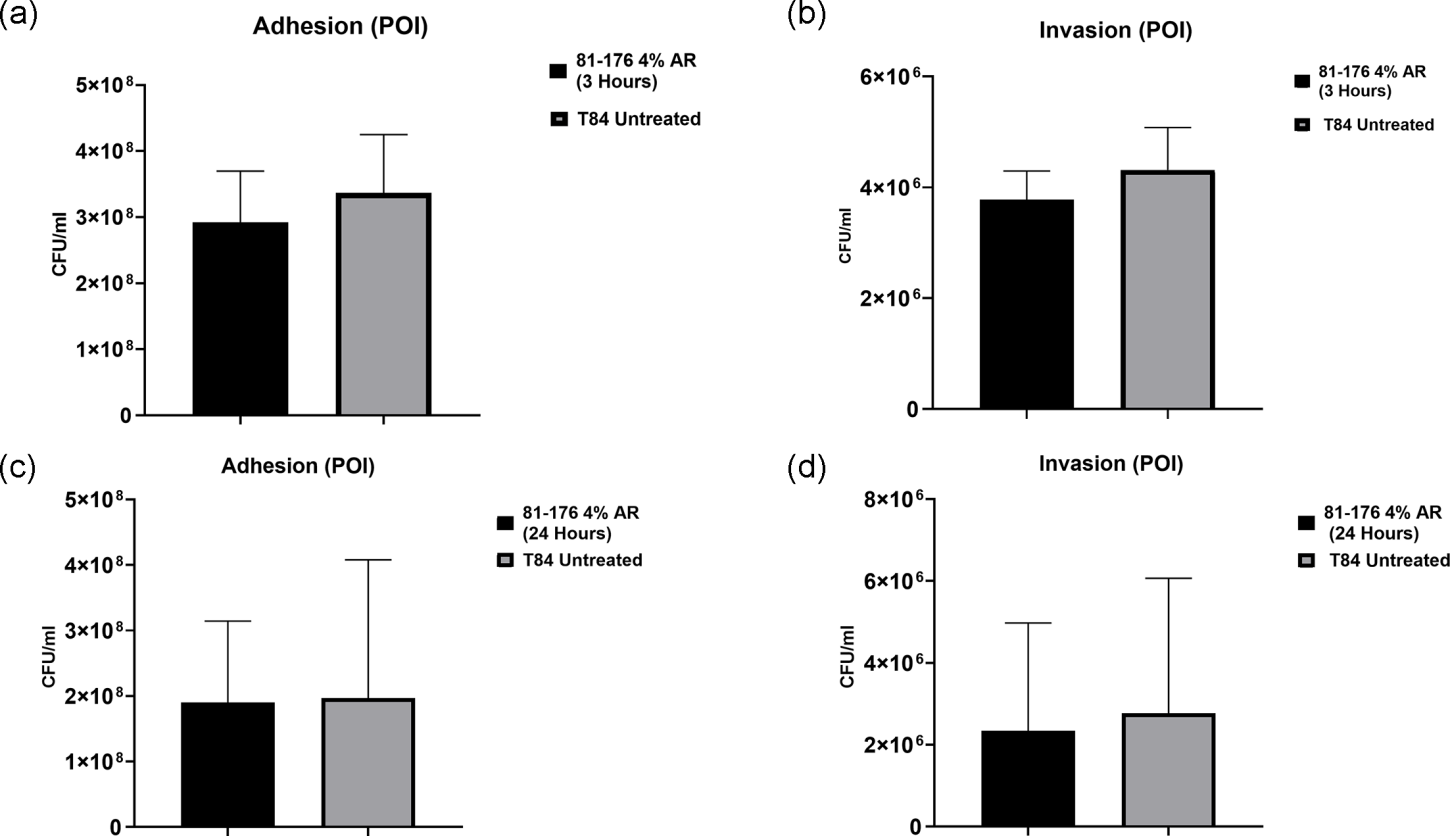
The effect of arrowroot on *C. jejuni* strain 81–176 interaction and invasion. T84 cells were exposed to arrowroot at the POI with *C. jejuni* for 3 and 24 h. (**a, c**) T84 cells were washed with PBS, lysed and the numbers of interacting bacteria were assessed. (**b, d**) For invasion assay, after infection with *C. jejuni*, IECs were incubated with gentamicin (150 µg ml^−1^) for 2 h to kill extracellular bacteria, then lysed and the numbers of intracellular bacteria were assessed. Experiments were repeated in three biological and three technical replicates. Unpaired t-tests were used to compare two datasets for significance. Data are presented as mean±sem.

**Fig. 4.**
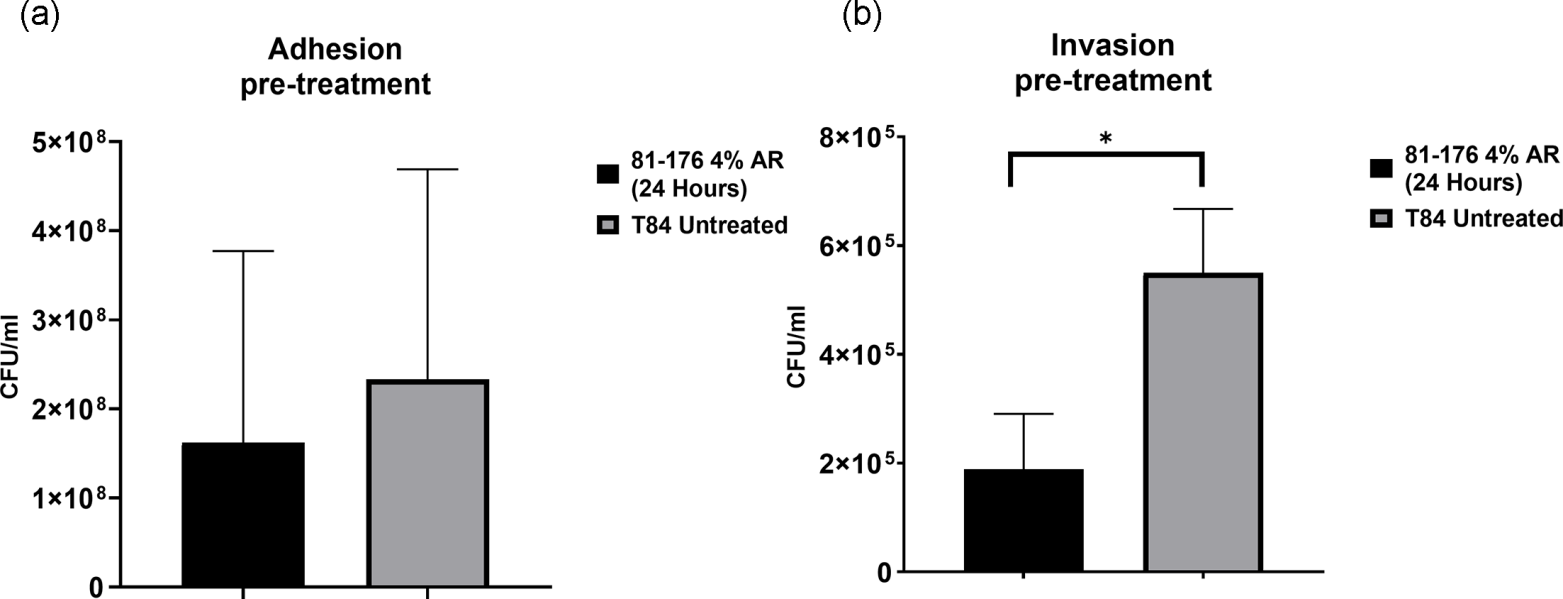
The effect of arrowroot on *C. jejuni* strain 81–176 interaction and invasion. T84 cells were pretreated with 4% concentration of arrowroot for 24 h and infected with *C. jejuni* for 3 h. (**a**) T84 cells were washed with PBS, lysed and the numbers of interacting bacteria were assessed. (**b**) For the invasion assay, after infection with *C. jejuni*, IECs were incubated with gentamicin (150 µg ml^−1^) for 2 h to kill extracellular bacteria, then lysed and the numbers of intracellular bacteria were assessed. Experiments were repeated in three biological and three technical replicates. Unpaired t-tests were used to compare two datasets for significance. Data are presented as mean±sem. Asterisks denote a statistically significant difference (* = P<0.05).

The interaction and invasion of T84 cells by the highly invasive *C. jejuni* strain 81–176 were examined with and without arrowroot to assess its impact on pathogenicity. Using *in vitro* infection assays, as outlined in Materials and Methods (Section 2.5), we identified no significant differences in interaction, invasion or intracellular survival between untreated T84 cells and those exposed to arrowroot, whether introduced before (Fig. 3) or at the POI. However, a notable exception was observed: a significant reduction (* = P<0.05) in invasion occurred when T84 cells were pretreated with a 4% arrowroot concentration for 24 h (Fig. 4). Some data were not included in the figures. Specifically, results for the effect of arrowroot on *C. jejuni* strain 81–176 interaction and invasion in T84 cells pretreated for 1 and 3 h were excluded, as these differences were not statistically significant. Similarly, intracellular survival data were excluded because no significant differences were observed between treated and untreated conditions.

### IL-8 ELISA

IL-8 induction by *C. jejuni* strain 81–176 was measured to investigate whether *C. jejuni* interaction and invasion are correlated to *C. jejuni*-induced IL-8 secretion by T84 cells. To determine IL-8 induction in T84 cells after 3 and 24 h of introduction to a 4% concentration of arrowroot at the time of infection by *C. jejuni* strain 81–176, human IL-8 ELISA was performed. There were no significant differences in IL-8 induction between T84 cells infected by *C. jejuni* strain 81–176 compared with IL-8 induction in T84 cells after 3 and 24 h of introduction to a 4% concentration of arrowroot. The observations indicate that arrowroot did not reduce *C. jejuni* strain 81–176 induction of IL-8 in T84 cells at the time of infection at the selected concentration.

**Fig. 5.**
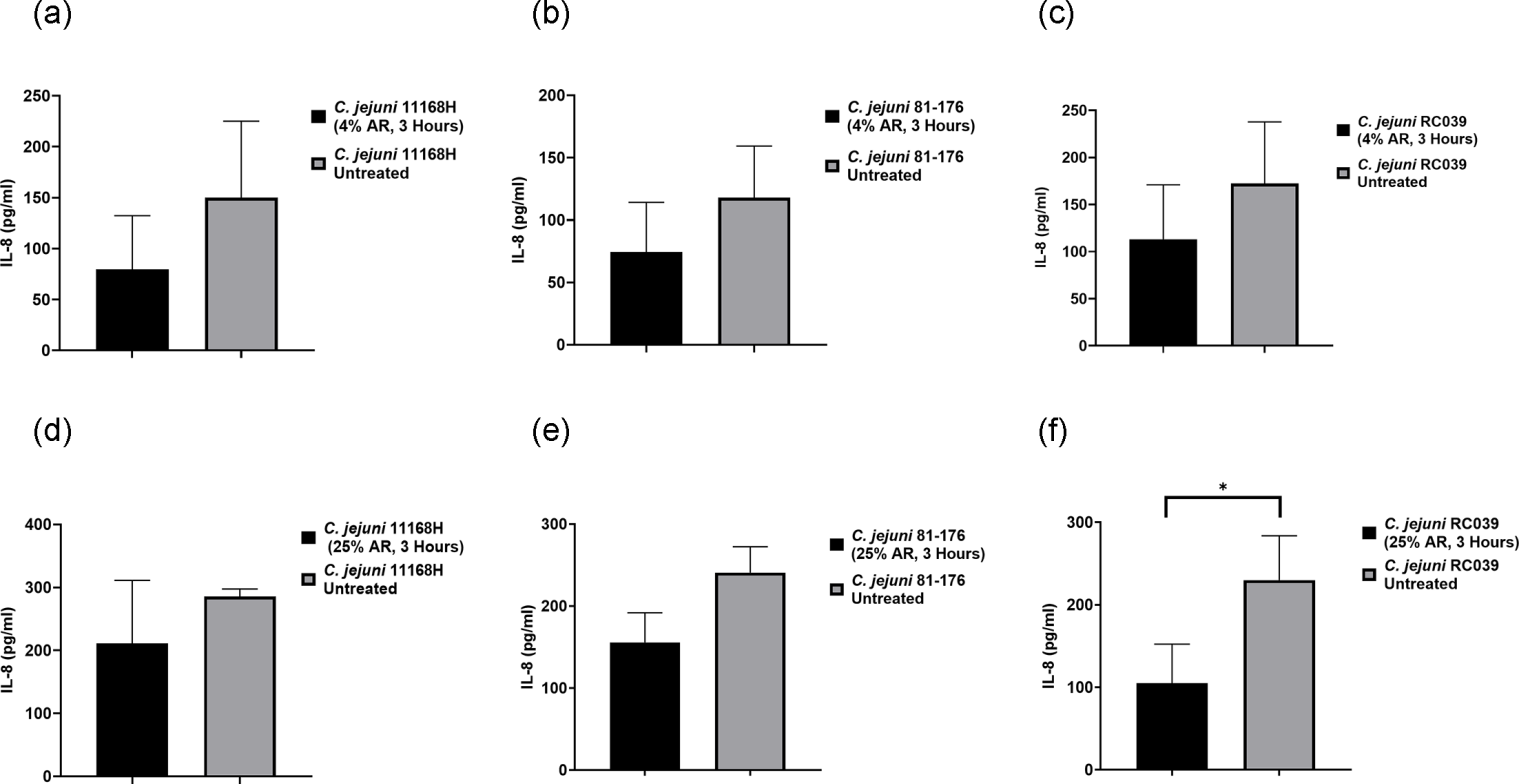
IL-8 release from T84 cells infected with *C. jejuni* 11168H, 81–176 and RC039 strains. T84 cells in a 24-well plate were infected with *C. jejuni* for 3 h at 37 °C in a 5% CO_2_ incubator (MOI of 200:1). (**a–c**) T84 cells were treated with 4% concentration of AR for 3 h, (**d–f**) treated with 25% concentration of AR for 3 h. Medium from each well was subjected to human IL-8 ELISA to measure the concentrations of IL-8. Three biological and two technical replicates were performed for each experiment. Unpaired t-tests were used to compare two datasets for significance. Data are presented as mean±sem. Asterisks denote a statistically significant difference (* = P<0.05). AR, arrowroot.

Furthermore, the pro-inflammatory response of T84 cells was investigated by measuring the expression patterns of the cytokine IL-8 released from T84 cells infected for 3 h with various bacterial strains (*C. jejuni* 11168H, 81–176, RC039, *C. coli* C75, M8 and *E. coli*). Following this, the T84 cells were treated with 4% and 25% arrowroot for 3 h. While there was a reduction in the cytokine IL-8 released from T84 cells infected with the bacterial strains (*C. jejuni* 11168H, 81–176, RC039) compared with IL-8 induction in T84 cells after 3 h of treatment with 4% and 25% concentrations of arrowroot, the reduction was not statistically significant, except for *C. jejuni* strain RC039 treated with a 25% concentration of arrowroot (Fig. 5).

**Fig. 6.**
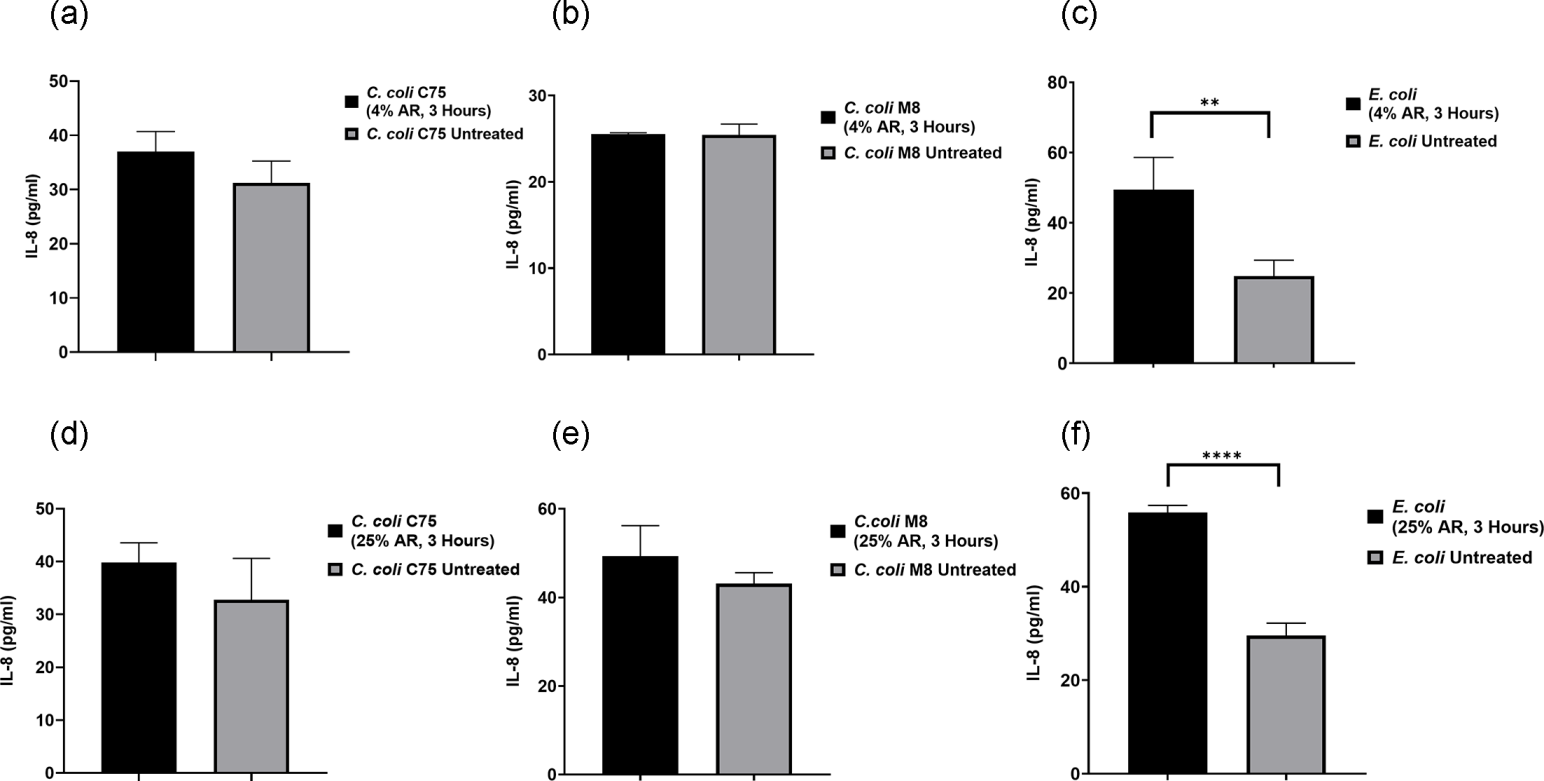
IL-8 release from T84 cells infected with *C. coli* C75, M8 strains and *E. coli*. T84 cells in a 24-well plate were infected with bacterial strains for 3 h at 37 °C in a 5% CO_2_ incubator (MOI of 200:1). (**a–c**) T84 cells were treated with 4% concentration of AR for 3 h, (**d–f**) treated with 25% concentration of AR for 3 h. Medium from each well was subjected to human IL-8 ELISA to measure the concentrations of IL-8. Three biological and two technical replicates were performed for each experiment. Unpaired t-tests were used to compare two datasets for significance. Data are presented as mean±sem. Asterisks denote a statistically significant difference (** = P<0.01; **** = P<0.0001). AR, arrowroot.

There was no reduction in the cytokine IL-8 released from T84 cells infected with the bacterial strains (*C. coli* C75 and *C. coli* M8) compared with IL-8 induction in T84 cells after 3 h of treatment with 4% and 25% concentrations of arrowroot; however, there was an increase of IL-8 release when cells were infected with *E. coli* at both concentrations of arrowroot (Fig. 6). To determine whether arrowroot alone modulates IL-8 production, we assessed cytokine levels in T84 cells incubated with 4% and 25% arrowroot in the absence of bacteria. IL-8 levels remained comparable to baseline (media-only controls), suggesting that arrowroot does not independently stimulate IL-8 secretion. These controls confirm that the observed cytokine responses in Figs 5 and 6 are attributable to bacterial stimulation and its modulation by arrowroot.

### Cytotoxicity of selected concentrations of arrowroot on T84 cells and Trypan blue exclusion assay

**Fig. 7.**
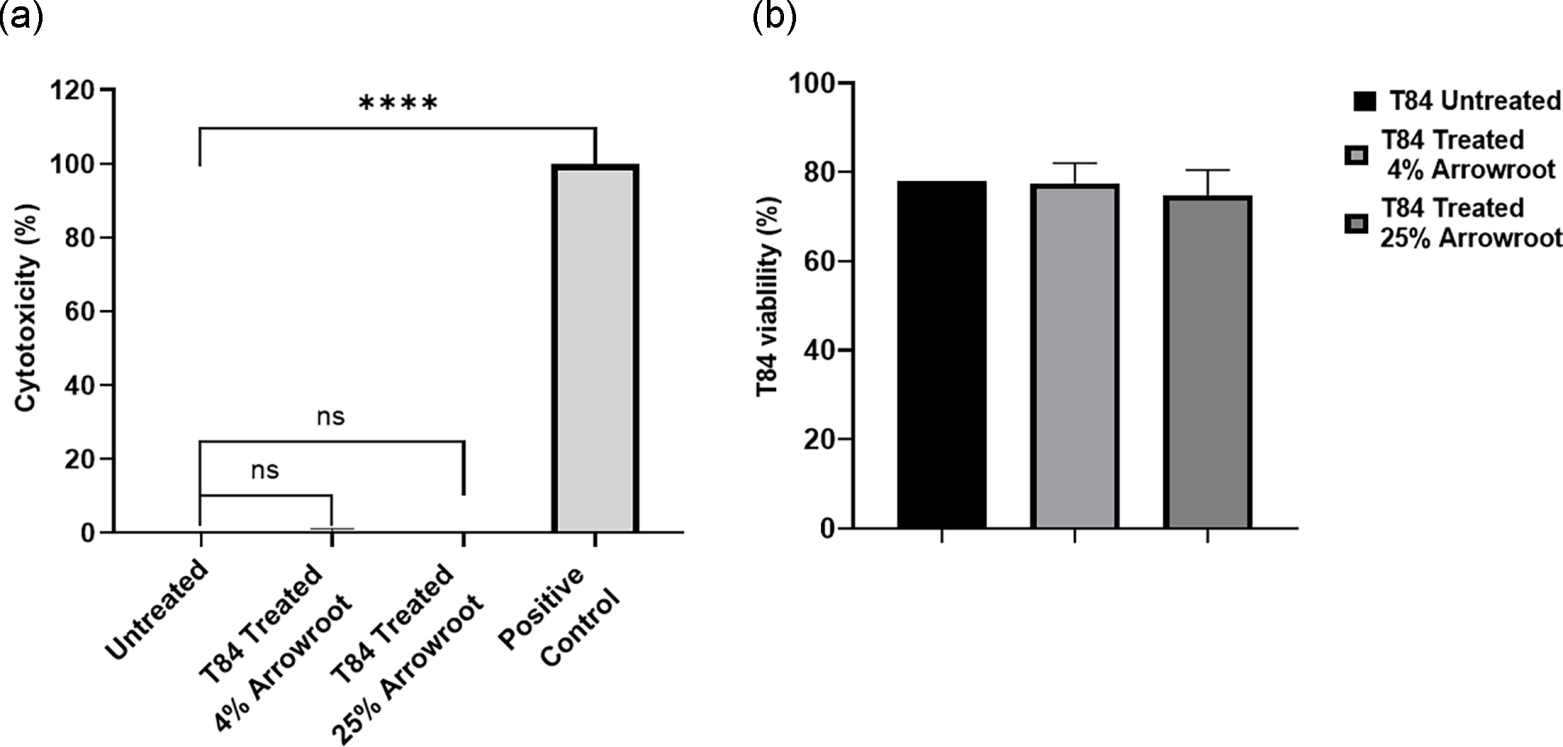
Measurement of cytotoxicity of different concentrations of arrowroot on T84 cells. (**a**) T84 cells grown in a 96-well plate were incubated at 37 °C in a 5% CO2 incubator. Medium from each well was then analysed using a LDH assay. Cytotoxicity (%) was calculated as follows: (treated LDH activity – spontaneous LDH activity)/(maximum LDH activity – spontaneous LDH activity) × 100. Two biological and three technical replicates were performed for each experiment. Unpaired t-tests were used for comparison. Data are presented as mean±sem. Asterisks denote a statistically significant difference (**** = *P*<0.0001). (**b**) Trypan blue exclusion assay. T84 cells were treated with 4% and 25% concentrations of arrowroot for 3 h. Percentage viability was calculated based on the enumerated cells.

To determine whether the selected concentrations of arrowroot exhibit any cytotoxicity on T84 cells, an LDH assay was conducted. The results showed that the 4% and 25% concentrations of arrowroot did not exhibit cytotoxicity or differences in cytotoxicity on T84 cells when compared with untreated cells. A highly significant difference was observed between the untreated cells and the positive control (**** = *P*<0.0001), which served as the reference for 100% cytotoxicity, validating the assay and enabling data normalization. These observations indicate that the use of arrowroot at the selected concentrations is safe (Fig. 7a). To determine the impact of the selected concentrations on T84 cell viability, a trypan blue exclusion assay was performed; similar viability percentages were observed in untreated and treated T84 cells (Fig. 7b). The arrowroot extract was visually colourless and did not affect LDH or TMB absorbance values, confirming that the extract did not introduce optical interference during the assays. Raw data are provided in Supplementary Data S1 and Data S2 (available in the online Supplementary Material).

## Discussions

Reducing *Campylobacter* infection in chicken products is essential to ensure food safety. *Campylobacter* remains a significant microbiological contaminant in chicken products [33]. New antimicrobials are important for controlling infection and multidrug-resistant strains of *Campylobacter.* Natural antimicrobials have emerged as a promising alternative for pathogen reduction [24], particularly by targeting bacterial virulence. For instance, research by van Alphen *et al.* [34] suggests that carvacrol, the primary component of oregano, significantly diminishes the virulence potential of *C. jejuni*, offering protection against cellular infection in intestine 407 (INT-407) cells [34,36]. Similarly, another study indicates that Citral, a compound found in citrus extracts, effectively suppresses *C. jejuni* attachment and invasion in Caco-2 cells [37]. It was shown that carvacrol disrupts bacterial outer membranes [38,39], leading to increased permeability and cell damage. This mechanism is comparable with medium-chain fatty acids, which compromise cell integrity [40,42], and short-chain fatty acids, which interfere with intracellular processes after membrane diffusion [43,45].

Studies have highlighted the effectiveness of carvacrol when combined with other antimicrobial agents. For instance, a blend of propionic acid, sorbic acid, thymol and eugenol has demonstrated significant reductions in *Campylobacter* populations in animal models [45]. Similarly, oregano and lactic acid have shown synergistic antibacterial activity *in vitro*, reinforcing the potential of combined antimicrobial approaches [46]. Additionally, another study indicates that Citral, a compound found in citrus extracts, effectively suppresses *C. jejuni* attachment and invasion in Caco-2 cells. These findings highlight the potential of plant-derived compounds as natural interventions to mitigate foodborne pathogens, offering an alternative to conventional antimicrobial strategies. Arrowroot has long been valued for its therapeutic properties, particularly in addressing digestive issues like diarrhoea and dysentery. Traditionally, it has been used as a soothing agent due to its easily digestible starch, which can help calm gastrointestinal irritation and support recovery [47]. Arrowroot is highly digestible and gluten-free and, consequently, can be administered to patients with digestive issues or coeliac disease [48]. Studies have shown that arrowroot has antimicrobial properties against pathogenic bacteria, making it an alternative to antibiotics, especially for antibiotic-resistant pathogens like the Gram-positive bacterium *Staphylococcus aureus*. The MIC and MBC against strain methicillin-resistant *S. aureus* were determined to be 100%, with a mean inhibitory zone diameter of 15.5 mm [49]. No studies have been done to evaluate its effectiveness against *C. jejuni* and *C. coli.* Therefore, we assessed the antibacterial effect of arrowroot on reducing the numbers of different strains of *C. jejuni*, *C. coli* and *E. coli*. Our results indicated that 4% and 25% concentrations significantly reduced bacterial growth in *C. jejuni* strains. While these concentrations were effective in reducing bacterial growth in *C. coli* strains, the results were not statistically significant. However, a 25% concentration was statistically significant in reducing *E. coli*. We hypothesize that the ability to reduce bacterial growth might be due to the antioxidant activity of the phenolic compounds in arrowroot. Adhesion is a key bacterial virulence factor for colonization, and reducing this characteristic may minimize *Campylobacter*’s ability to attach to epithelial cells. Previous studies by Byrne *et al.* [50] observed similar efficiencies in *Campylobacter* spp*.* attachment and invasion of both primary chicken enterocytes and human epithelial cells [50]. Conversely, research by Willer *et al.* [51] highlighted evident differences between poultry and human gut cells in their interactions with *C. jejuni* [51]. Several studies have investigated the effects of various natural antimicrobials on *Campylobacter* spp. adhesion and invasion. Auranta 3001 has demonstrated the ability to reduce the pathogenic properties of Type VI secretion system (T6SS) *Campylobacter in vitro*, as well as decrease colonization *in vivo*. Moreover, this reduction is associated with a significant decrease in bacterial motility in the studied *Campylobacter* species [52]. Other studies have explored the antimicrobial potential of natural extracts, such as Blackberry (*Rubus fruticosus*) and Blueberry (*Vaccinium corymbosum*), revealing their ability to significantly reduce bacterial adhesion and invasion of epithelial cells. Similarly, extracts from *Artemisia ludoviciana*, *Acacia farnesiana*, *Cynara scolymus* spp., *Opuntia ficus-indica* and β-resorcylic acid have exhibited comparable effects, suggesting their promising role in controlling pathogenic bacteria. These findings highlight the potential of plant-based compounds as effective alternatives for mitigating bacterial infections while reducing reliance on synthetic antimicrobials [53,54]. To explore the antimicrobial ability of arrowroot, we examined its potential to inhibit the invasiveness of epithelial cells, focusing on *C. jejuni* 81–176 strain. However, our results indicated that a 4% concentration of arrowroot, tested at various intervals (1, 3 and 24 h), was ineffective in reducing the invasiveness of the 81–176 strain. Additionally, we assessed IL-8 induction by *C. jejuni*, further evaluating its interaction with epithelial cells. To assess IL-8 induction in T84 cells after 3 and 24 h of exposure to a 4% concentration of arrowroot during infection with *C. jejuni* strain 81–176, a human IL-8 ELISA assay was conducted. The results indicated that arrowroot did not reduce IL-8 induction by *C. jejuni* strain 81–176 in T84 cells at the selected concentration during the infection period. This outcome may be attributed to the concentration of arrowroot and the duration of exposure, suggesting that arrowroot might require a higher concentration and a longer time to employ its effects. To explore the anti-inflammatory potential of arrowroot on cytokine IL-8 release, our results revealed a reduction in IL-8 levels from T84 cells infected with *C. jejuni* strains (11168H, 81–176, RC039) following 3 h of treatment with 4% and 25% concentrations of arrowroot. However, this reduction was not statistically significant, except for *C. jejuni* strain RC039, where a 25% arrowroot concentration led to a statistically significant decrease in IL-8 release (Fig. 4).

Conversely, no reduction in IL-8 release was observed for T84 cells infected with *C. coli* strains (C75 and M8) under similar conditions. Notably, *E. coli*-infected T84 cells exhibited an increase in IL-8 release at both 4% and 25% concentrations of arrowroot, indicating a differential response (Fig. 5). IL-8 responses vary significantly between *E. coli* and *C. jejuni*, reflecting distinct host–pathogen interaction mechanisms. *E. coli*, especially pathogenic strains such as Enteropathogenic *E. coli* (EPEC) and Enterohemorrhagic *E. coli* (EHEC), typically induces strong IL-8 secretion via TLR4 recognition of lipopolysaccharide, leading to NF-κB activation and robust neutrophil recruitment [55]. In contrast, *C. jejuni* elicits more variable IL-8 responses, influenced by its unique lipooligosaccharide, flagellin structure and intracellular invasion capacity [56,57]. These differences may be attributed to the absence of classical secretion systems in *C. jejuni* and its reliance on host cell signalling modulation [55]. This could suggest a unique interaction between arrowroot and *E. coli* that requires further investigation.

## Conclusions

The study demonstrated that 4% and 25% concentrations of arrowroot extract were non-cytotoxic to T84 cells and effectively reduced bacterial growth in *C. jejuni* strains (11168H, 81–176, RC039). The extract also inhibited the growth of *C. coli* (C75 and M8) and *E. coli*, with statistically significant results against *E. coli* at the 25% concentration. These findings highlight the potential of arrowroot extract as a natural alternative to combat bacterial resistance and improve food safety.

To build on the current findings, future investigations will incorporate a broader collection of assays to elucidate the mechanistic and functional impacts of the compound. These include membrane integrity assays (e.g. propidium iodide uptake), motility assays, biofilm formation and quorum sensing evaluations and time–kill kinetics. Chemical characterization using HPLC and gas chromatography–mass spectrometry (GC-MS) will further define active constituents. Importantly, we intend to include a comparative compound in subsequent studies to strengthen interpretability and contextualize the observed effects within a broader antimicrobial and immunomodulatory framework. Further exploration of the mechanisms behind arrowroot’s effects, and conducting trials to evaluate its safety and efficacy in live chicken models, could provide crucial insights into its broader applications and impactful advancements in addressing antibiotic resistance and improving food safety on a larger scale.

## Supplementary material

10.1099/mic.0.001658 Uncited Supplementary Material 1.

10.1099/mic.0.001658 Uncited Supplementary Material 2.

## Abbreviations

CBA: Columbia Blood Agar
DMEM/F-12: Dulbecco’s modified Eagle’s medium and Ham’s F-12 medium
ECACC: European Collection of Authenticated Cell Cultures
EU: European Union
EUCAST: European Committee on Antimicrobial Susceptibility Testing
IEC: intestinal epithelial cell
IL-8: interleukin-8
LDH: lactate dehydrogenase
MBC: minimum bactericidal concentration
MHB: Müeller–Hinton broth
MOI: multiplicity of infection
OD_600_: OD at 600 nm
POI: point of infection
TMB: Tetramethylbenzidine
WHO: World Health Organization
